# The Psychosocial Resonance of Food Safety Risk: A Space-Time Perspective

**DOI:** 10.3390/foods14132260

**Published:** 2025-06-26

**Authors:** Lei Wang, Han Sun, Tingqiang Chen

**Affiliations:** School of Economics and Management, Nanjing Tech University, Nanjing 211816, China; leiwang19900820@njtech.edu.cn (L.W.);

**Keywords:** space-time perspective, food safety risk, psychosocial resonance, cellular automata

## Abstract

From a space-time perspective, this paper constructs a CA-SHIRS model to study the psychosocial resonance diffusion of food safety risk, using complex network and cellular automata theory. The CA-SHIRS model is a framework that combines cellular automata with SHIRS (Susceptible–Hidden–Infected–Recovered–Susceptible) epidemic modeling. This methodological integration can effectively reflect local interactions and spatial distribution among consumers. Furthermore, this paper analyzes the diffusion mechanism and spatial–temporal evolution of the psychosocial resonance of food safety risk, considering the interaction between consumer heterogeneity and media communication strategies. The primary conclusions are outlined as follows: (1) An increase in infection probability, conversion probability, and immune failure probability causes the psychosocial resonance of food safety risk to spread rapidly across different regions and populations. In contrast, an increase in immune probability helps control the psychosocial resonance of food safety risk. (2) The diffusion threshold of the psychosocial resonance of food safety risk is negatively related to the consumer risk perception level, consumer risk attention, media freedom, and media report authenticity. However, it is positively correlated with consumer sentiment, market noise, and media report tendency. (3) The consumer risk perception level, consumer risk attention, media freedom, and media report authenticity can effectively inhibit the spatial–temporal diffusion of the psychosocial resonance of food safety risk. On the other hand, increases in market noise, consumer sentiment, and media report tendency accelerate the spread of the psychosocial resonance of food safety risk across different regions.

## 1. Introduction

Food safety has been a global concern for a long time [1]. Some severe food safety events, such as the Salmonella contamination of infant milk powder in Europe in 2017, not only endanger consumers’ health and lives but also spread rapidly across different temporal and spatial dimensions, severely disrupting market order and triggering widespread public concern and anxiety. In recent years, with the increasing diversification of information transmission channels, the spatial–temporal evolution characteristics of food safety risk have become more complex, especially due to the fast development of various social networks and media platforms. This is reflected in the gradual expansion of food safety risk from local areas to the national and even global scope. Moreover, public emotions and psychology exhibit the resonance effect across regions, time, and populations during the dissemination of food safety incidents. Under such circumstances, the psychosocial resonance of food safety risk emerges and propagates persistently, posing severe threats to food market integrity and social order. From a space-time perspective, the psychosocial resonance of food safety risk refers to the mutual influence of individuals’ and groups’ cognitive, emotional, and behavioral responses to food safety risk information within a specific time and spatial context following a food safety incident. Additionally, this phenomenon is affected not only by individual factors of consumers but also by psychosocial factors such as social connectedness and media communication [2]. Therefore, it is of great practical significance to study the diffusion mechanism and spatial–temporal evolution of the psychosocial resonance of food safety risk from a space-time perspective to maintain the stability of the food market and social order.

Currently, research on food safety risk is extensive, with scholars examining the issue from various perspectives, including government regulation [3,4,5,6], consumer risk perception [7,8], and media communication [9,10]. Existing studies have highlighted the food industry’s shift towards multi-format decentralized management and large-scale multi-channel circulation [11,12]. In particular, the cross-regional transmission of food safety risk has become more evident, posing greater challenges to food regulation and the ecosystem [13,14]. Furthermore, during the cross-regional diffusion of food safety risk, psychosocial resonance exacerbates the impact and scope of food safety incidents [15,16]. Meanwhile, news reports and social media can also accelerate the spread of negative psychological states such as panic and anxiety among consumers. As time goes on, this can trigger mass panic, forming the psychosocial resonance of food safety risk [17]. However, existing studies mostly focus on either a time or space perspective or only consider the influence of single factors, such as consumer behavior or media communication strategies. For example, while Li et al. (2023) and Nie and Liu (2023) offered valuable insights into the spatial patterns of cross-regional food safety risk transfer, their analytical frameworks did not explicitly explore temporal dynamics [13,18]. Again, Zhu et al. (2021) and Zhang et al. (2022) offered significant contributions from consumer behavior and media communication perspectives, respectively [7,9], but the synergistic effects arising from their interaction remain underexplored. The psychosocial resonance of food safety risk is reflected not only in the risk perceptions and emotional responses of individual consumers but also in the dynamic interactions of group psychology across time and regions [19,20]. This resonance breaks the boundaries of time and space as information dissemination speeds up and interpersonal networks expand, causing significant adverse impacts on the food supply chain and the entire food market [21,22]. Therefore, to resolve fundamental limitations in prior work, this paper constructs a CA-SHIRS model to analyze the spatial–temporal evolution characteristics of the social psychological resonance diffusion of food safety risk from a space-time perspective, under the interaction of consumer heterogeneity and media communication strategies. Critically, this study advances prior research through two fundamental breakthroughs. It pioneers a spatial–temporal integration framework by embedding geographic constraints and real-time dynamics into cellular automata transition rules. Concurrently, it formalizes and quantifies the synergistic amplification between consumer heterogeneity and media communication strategies. This is the first innovation.

Epidemic models based on complex networks have been applied in various fields, including public health [23,24], information communication [25,26], financial risk contagion [27,28], and food safety risk diffusion [29,30,31], among others. For instance, Khare et al. [31] used an epidemic model to analyze the impact of food adulteration on consumers’ health under the influence of media communication, conducting simulations to explore the spread and evolutionary characteristics of diseases caused by food adulteration. Currently, the application of epidemic models in food safety risk diffusion mainly focuses on conventional models such as SI (Susceptible–Infected), SIS (Susceptible–Infected–Susceptible), and SIRS (Susceptible–Infected–Recovered–Susceptible). However, there have been limited explorations of two critical aspects: the dynamic evolution of consumer states during psychosocial resonance diffusion processes and the complex interactions among consumers in different states. The psychosocial resonance diffusion of food safety risk demonstrates distinctive spatial–temporal patterns. Meanwhile, cellular automata (CA), a grid-based dynamic model that discretizes time, space, and states, effectively captures these spatial–temporal evolution characteristics. CA can simulate the dynamic processes of complex systems using specific state transition rules [32,33]. Therefore, combining cellular automata with an epidemic model offers a more comprehensive approach to studying the spatial–temporal evolution of the psychosocial resonance of food safety risk. Unlike conventional epidemic models that abstract space into homogeneous networks, combining cellular automata with an epidemic model can embed geographically grounded cellular automata grids that explicitly simulate distance decay and neighborhood contagion effects. Building on this conceptual framework, the present study proposes an optimized diffusion rule for the psychosocial resonance of food safety risk by integrating the SHIRS epidemic model with cellular automata theory. This not only enhances the traditional epidemic model but also accounts for the multi-level interactions and feedback of psychosocial resonance in the spatial–temporal dimension. This is the second innovation.

In summary, this paper constructs a CA-SHIRS model to study the psychosocial resonance diffusion of food safety risk from a space-time perspective, considering the interaction of consumer heterogeneity and media communication strategies. It then conducts numerical simulations to analyze the spatial–temporal evolution characteristics of the psychosocial resonance diffusion of food safety risk. The main contributions of this paper are as follows: (1) Unlike existing studies that focus solely on either a temporal or spatial perspective or consider only single factors such as consumer behavior or media communication, this paper analyzes the evolution characteristics of the psychosocial resonance diffusion of food safety risk from a space-time perspective, incorporating the interaction between consumer heterogeneity and media communication strategies. (2) Unlike conventional epidemic models, this study incorporates cellular automata theory into the SHIRS epidemic model. This approach not only optimizes the diffusion rule of the psychosocial resonance of food safety risk but also constructs a CA-SHIRS model that better reflects local interactions and spatial distribution among consumers. (3) This paper presents several innovative and practically valuable conclusions: The consumer risk perception level, consumer risk attention, media freedom, and media report authenticity can effectively suppress the spatial–temporal diffusion of the psychosocial resonance of food safety risk. An increase in market noise, consumer sentiment, and media report tendency lead to the rapid diffusion of the psychosocial resonance of food safety risk across different regions. Notably, the risk amplification effect of media report tendency is significantly stronger than the combined risk mitigation effect of media report authenticity and media freedom.

The structure of this article is as follows. Section 2.1 introduces the diffusion mechanism of the psychosocial resonance of food safety risk. Section 2.2 presents the CA-SHIRS model of the psychosocial resonance diffusion of food safety risk. Section 3 provides the simulation results and analysis. Finally, Section 4 offers conclusions.

## 2. Materials and Methods

### 2.1. Diffusion Mechanism of Psychosocial Resonance of Food Safety Risk

**Figure 1:**
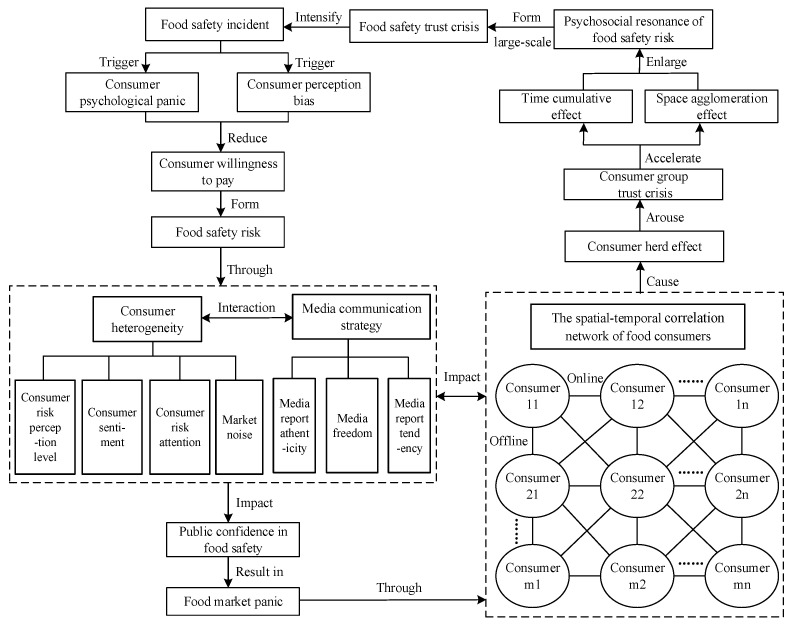
Diffusion mechanism of psychosocial resonance of food safety risk under interaction of consumer heterogeneity and media communication strategies.

The psychosocial resonance of food safety risk can result in a sharp and synchronized change in social psychology, causing a psychological shockwave throughout the entire food market [15,34]. This leads to the formation of a shared perception and emotional resonance of food safety risk, which in turn triggers a rapid psychological change within the group. Through continuous accumulation and rapid diffusion, the psychosocial resonance of food safety risk will exhibit different spatial–temporal evolution characteristics, influenced by varying consumer heterogeneity factors. Consumer heterogeneity encompasses four key variables: consumer risk perception level, consumer risk attention, consumer sentiment, and market noise. Among them, the consumer risk perception level refers to the consumer’s ability to recognize food safety risks; consumer risk attention denotes the degree of attention consumers devote to food safety risk information; consumer sentiment refers to the consumer’s emotional state, which is divided into positive, negative, and neutral; and market noise represents the degree of environmental information interference. At the same time, the intensity and scope of the psychosocial resonance diffusion of food safety risk vary under different media communication strategies, thereby exhibiting different spatial–temporal patterns. This paper illustrates the diffusion mechanism of the psychosocial resonance of food safety risk under the interaction of consumer heterogeneity and media communication strategies, as shown in Figure 1. Figure 1 reveals how food safety risks trigger group panic and erode public trust through the synergistic interaction between consumer heterogeneity and media communication strategies. This group panic subsequently evolves into psychosocial resonance within the spatial–temporal correlation network of food consumers, where such resonance propagates through these interconnected systems. This figure’s core contribution lies in deconstructing both the emergence mechanism and diffusion dynamics of the psychosocial resonance of food safety risk, thereby establishing the theoretical groundwork for subsequent methodological modeling.

Firstly, this paragraph specifically introduces how food safety risks affect group psychology and public trust through consumer heterogeneity and media communication strategies. Some food enterprises may engage in speculative behaviors, driven by factors such as market information asymmetry, extended supply chains, and structural market shifts [35]. These behaviors can lead to irrational decision-making and ultimately result in food safety incidents. Meanwhile, consumers are not completely rational when confronted with food safety incidents. After the outbreak of food safety incidents, consumer panic may arise, leading to biases in consumers’ risk perception that decrease consumers’ willingness to purchase. As a result, food safety risk exhibits a diffusion pattern across regions and populations. On the one hand, the media will publicize and comment on breaking food safety incidents. If media coverage is inaccurate, media freedom is constrained, or if the media primarily reports negative information, this will exacerbate panic in the food market and undermine public confidence in food safety. When consumers are influenced by negative food safety incidents, their subjective perceptions may lead them to develop a negative attitude toward the entire food market. On the other hand, some consumers who have a low level of risk perception, who are easily swayed by emotions, or who pay less attention to risks may find it difficult to objectively assess sudden food safety incidents, leading to cognitive and behavioral biases. In this situation, public confidence in food safety significantly declines, causing panic in the market. At this point, panic in the food market spreads both temporally and spatially, further exacerbating the concerns of food safety risk.

Secondly, this paragraph specifically analyzes how group panic evolves into the psychosocial resonance of food safety risk through the spatial–temporal correlation network of food consumers and how psychosocial resonance further spreads in the spatial–temporal correlation network of food consumers. Due to the interconnectedness of consumers in the food market, consumers may be influenced not only by their own preferences and media communication strategy but also by the behavior of other consumers in the market. A consumer is often embedded in various interpersonal relationships, forming a multi-level network structure through family ties, work relationships, online communication, and more. Once a consumer in the network encounters food safety risk, information can easily spread to surrounding consumers through online communication or offline conversations within the spatial–temporal correlation network of food consumers. Under the influence of the herd effect, this can trigger a trust crisis within the spatial–temporal correlation network of food consumers and even within the entire consumer group. As a result, the psychosocial resonance of food safety risk will continuously accumulate over time and space, gradually expanding from localized areas to the entire population. Ultimately, this can trigger a widespread food safety trust crisis, further intensifying the impact and scope of food safety incidents.

### 2.2. CA-SHIRS Model of Psychosocial Resonance Diffusion of Food Safety Risk

#### 2.2.1. Cellular State Setting

The cellular automata model is composed of cellular space, a cell, a neighborhood, and a dynamical evolution rule, which is usually represented by a tetrad $CA=(\Omega_{d},C,N,F)$. Here, $\Omega_{d}$ is a d-dimensional cellular space, a discrete spatial grid collection, usually two-dimensional. C represents the state of cells, which is influenced by its own state and the states of neighboring cells. N is the neighborhood of cells, the set of cells that influence the state of the central cell at the next time point. F is the evolution rule that determines the state change of cells at the next moment.

In this paper, consumers are represented as nodes in a complex network, with edges indicating the associative relationships formed through online and offline communications. Each node serves as a carrier of the psychosocial resonance of food safety risk, while the edges represent the pathways through which the psychosocial resonance of food safety risk spreads. In other words, psychosocial resonance spreads to the surrounding consumers randomly, so it is regarded as an undirected network for research.

The core characteristics of the Barabási–Albert (BA) network are growth and preferential attachment [36]. Specifically, as new individuals join the network, the network grows increasingly larger over time. New nodes connect to existing nodes with probabilities proportional to their current degree. Real-world social networks also exhibit these two characteristics. In practice, key opinion leaders (KOLs) such as celebrities and experts inherently attract more attention. Moreover, high-influence individuals in social networks consistently acquire new connections over time. Previous studies have shown that social networks are scale-free networks in the real world [37,38], and the degree distribution of scale-free networks follows the power law distribution [39]. Thus, many studies have applied scale-free networks to real-world social network research [40,41]. Therefore, in order to fit the nature and dynamic characteristics of the psychosocial resonance diffusion of food safety risk, this paper constructs a Barabási–Albert (BA) scale-free network as the cellular space of the psychosocial resonance diffusion of food safety risk, each node consumer is regarded as one cell, and other consumers with whom a consumer has communication links are regarded as the neighboring consumers of that consumer.

According to the state of consumers under the interaction of consumer heterogeneity and media communication strategies after the outbreak of food safety incidents, the nodes are classified into four categories with reference to the research of Wang et al. and Hassan et al. [42,43]:

(1) Susceptible consumers (S) are individuals who do not have psychosocial resonance for the time being. However, they are susceptible to the impact of infected consumers with psychosocial resonance under the influence of media communication strategies and consumer heterogeneity, and they may become transformed into consumers with psychosocial resonance.

(2) Hidden consumers (H) are individuals who have already been affected by food safety risk. They will change their psychological state over time until they become infected consumers.

(3) Infected consumers (I) are individuals who have been affected by food safety risk. They are extremely sensitive to food safety incidents and have the ability to spread psychosocial resonance.

(4) Recovered consumers (R) are individuals who have strong risk perception and emotional regulation. Meanwhile, they have better risk coping measures and are not affected by the impact of food safety incidents or infected consumers.

In this paper, s, h, i, and r denote the number of the aforementioned four states of consumers in the spatial–temporal correlation network of food consumers. N is the total quantity of consumers in the network, and k is the maximum degree of consumers in the network. $S_{k}(t)$, $H_{k}(t)$, $I_{k}(t)$, and $R_{k}(t)$, respectively, represent the densities of susceptible, hidden, infected, and recovered consumers with degree k at time t, and $S_{k}(t)+H_{k}(t)+I_{k}(t)+R_{k}(t)=1$ ($0\leq S_{k}(t),H_{k}(t),I_{k}(t),R_{k}(t)\leq 1$). At time t, it is assumed that the probability of connection between a susceptible consumer and an infected consumer is $\Theta_{1}(t)$.

#### 2.2.2. Dynamical Evolution Rule

In order to improve the conformity degree between the model and the psychosocial resonance diffusion of food safety risk in reality, referencing the studies by Pereira et al. and Mugnaine et al. [44,45], we organically integrate cellular automata and take into account the numerical impact of the number of infected neighbors on the infection rate. Cellular state set C is (0, 1, 2, 3), where 0 represents susceptible consumers, 1 represents hidden consumers, 2 represents infected consumers, and 3 represents recovered consumers. On the basis of the classic SHIRS model, the conversion rules among consumers of four states are expressed as follows considering the interaction of neighbors’ cellular states.

(1) When exposed to a food safety incident, if consumers’ neighboring nodes contain infected consumers, susceptible consumers will become infected with a probability $p=1-{\left(1-\tau \right)}^{\inf}$, transitioning from state S to H. Here, inf represents the number of infected consumers among the neighboring consumers. For instance, in real-world scenarios, when surrounding consumers widely disseminate panic-inducing information, the probability of individual consumers being psychologically influenced increases significantly. Under such circumstances, individuals become more susceptible to psychological infection.

(2) After the outbreak of a food safety incident, when media report authenticity and media freedom are low, hidden consumers transition to infected consumers with conversion probability δ [46,47] influenced by consumer risk perception level and consumer sentiment. During food safety incidents, the proliferation of distorted media reports or critical information gaps causes consumer panic to progressively accumulate. This escalating anxiety ultimately triggers mass panic among interconnected groups, resulting in the socio-psychological resonance of food safety risks.

(3) When media reports are authentic and free, and the consumer risk perception level and risk attention are high, infected consumers transition to recovered consumers with immune probability μ [46,47]. Conversely, when media reports lack authenticity and freedom, and the consumer risk perception level and risk attention are low, recovered consumers revert to susceptible consumers with immune failure probability γ [46,47]. When media outlets promptly and effectively disclose food safety information, consumers with higher cognitive levels disseminate factual information to those around them. This effectively mitigates the formation of collective panic. Conversely, inadequate media coverage may cause previously calmed consumers to revert to panic and anxiety due to emerging incidents, thereby continuing to influence their social circles.

**Figure 2:**
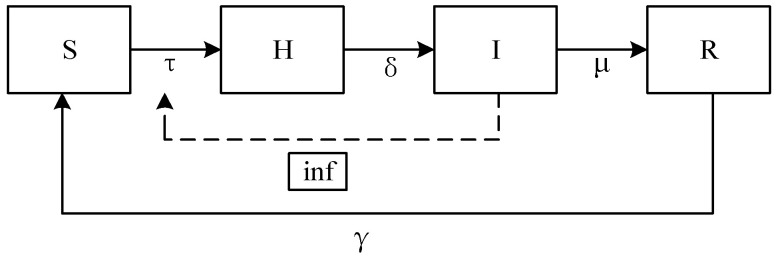
CA-SHIRS model of psychosocial resonance diffusion of food safety risk.

Based on the epidemic model set out above, this paper establishes the CA-SHIRS model of the psychosocial resonance diffusion of food safety risk, as shown in Figure 2.

In this paper, combining the Gilpin–Ayala information diffusion model and the behavioral effect function, and referring to the research of Wang et al. [48], the infection probability of the psychosocial resonance of food safety risk among consumers is further defined as follows.

(1) $$\tau =g(\sigma ,\phi ,\omega ,\varepsilon )f(\theta ,\rho ,\xi )=\frac{3}{2}\varepsilon^{2}e^{-2\sigma \omega }(1-e^{-2\phi })(1-e^{\frac{-\xi^{2}}{3\theta \rho }})$$

In this equation, $g(\sigma ,\phi ,\omega ,\varepsilon )=\frac{3}{2}\varepsilon e^{-2\sigma \omega }(1-e^{-2\phi })$ is the consumer heterogeneity function. σ (0 ≤ σ ≤ 1) is the consumer risk perception level [49,50,51]. A higher value of σ helps reduce negative emotions and improves consumers’ ability to make decisions in the face of food safety risk. ϕ (0 ≤ ϕ ≤ 1) is consumer sentiment [52,53,54]. ϕ approaches 1 for negative consumer sentiment, and ϕ=0.5 for smoother consumer sentiment, while ϕ approaches 0 for negative consumer sentiment, and negative sentiments tend to increase risk perception. ω (0 ≤ ω ≤ 1) is consumer risk attention [55,56]. A larger ω enhances consumers’ awareness of food safety risk, thereby mitigating the formation of adverse social psychology. ε (0 ≤ ε ≤ 1) is market noise [48,57], which has an impact on consumer behavior. In addition, $f(\theta ,\rho ,\xi )=\varepsilon (1-e^{\frac{-\xi^{2}}{3\theta \rho }})$ is the media communication strategy function. θ (0 ≤ θ ≤ 1) is media report authenticity [58,59]. The larger θ is, the better it can produce monitoring and deterrent effects on food enterprises and the more favorable to the formation of the social co-governance of food safety. ρ (0 ≤ ρ ≤ 1) is media freedom, and the larger ρ is, the more it can fulfill the role of media monitoring [60,61], which contributes to the detoxification of food safety risk and the mitigation of negative social psychology. ξ (0 ≤ ξ ≤ 1) is media report tendency [62,63]. ξ approaching 1 indicates that the media has a tendency to focus on negative reports. ξ=0.5 indicates that the media is inclined to report neutrally, and ξ approaching 0 indicates that the media has a tendency to focus on positive reports. The larger ξ is, the more likely it is to amplify food safety risk, leading to food safety panic and the formation of negative social psychology. The detailed explanations and benchmark values of the parameters involved in this article are shown in Table 1.

On the basis of the above analysis, according to the rules of state transition among consumers, the CA-SHIRS model of the social psychological resonance diffusion of food safety risk is as follows:

(2) $$\begin{array}{l} \left\{\begin{array}{l} \frac{dS_{k}(t)}{dt}=-k\tau S_{k}(t)\Theta (t)+\gamma R_{k}(t)\\ \frac{dH_{k}(t)}{dt}=k\tau S_{k}(t)\Theta (t)-\delta H_{k}(t)\\ \frac{dI_{k}(t)}{dt}=\delta H_{k}(t)-\mu I_{k}(t)\\ \frac{dR_{k}(t)}{dt}=\mu I_{k}(t)-\gamma R_{k}(t) \end{array}\right. \end{array}$$

#### 2.2.3. Threshold Analysis of Psychosocial Resonance Diffusion of Food Safety Risk

Making the right end of Equation (2) equal to 0, we can obtain the steady-state value of the density of susceptible, hidden, infected, and recovered consumers of degree k when the spatial–temporal correlation network of food consumers reaches the steady-state condition:

(3) $$\left\{\begin{array}{l} S_{k}^{*}(t)=\frac{\gamma \delta \mu }{\gamma k\delta \tau \Theta (t)+\gamma \delta \mu +\gamma k\mu \tau \Theta (t)+\delta \mu k\tau \Theta (t)}\\ H_{k}^{*}(t)=\frac{\gamma k\mu \tau \Theta (t)}{\gamma k\delta \tau \Theta (t)+\gamma \delta \mu +\gamma k\mu \tau \Theta (t)+\delta \mu k\tau \Theta (t)}\\ I_{k}^{*}(t)=\frac{\gamma \delta k\tau \Theta (t)}{\gamma k\delta \tau \Theta (t)+\gamma \delta \mu +\gamma k\mu \tau \Theta (t)+\delta \mu k\tau \Theta (t)}\\ R_{k}^{*}(t)=\frac{\delta k\mu \tau \Theta (t)}{\gamma k\delta \tau \Theta (t)+\gamma \delta \mu +\gamma k\mu \tau \Theta (t)+\delta \mu k\tau \Theta (t)} \end{array}\right.$$

Since $\Theta (t)=\frac{1}{<k>}\sum_{k}kP(k)I_{k}(t)$, <k> represents the average degree of the spatial–temporal correlation network of food consumers. From $<k>=\sum_{k}kP(k)$ and $<k^{2}>=\sum_{k}k^{2}P(k)$, Equation (4) is obtained.

(4) $$\Theta (t)=\frac{1}{<k>}\sum_{k}kP(k)\frac{\gamma \delta k\tau \Theta (t)}{\gamma k\delta \tau \Theta (t)+\gamma \delta \mu +\gamma k\mu \tau \Theta (t)+\delta \mu k\tau \Theta (t)}$$

Let Θ=Θ(t); then the above equation has a trivial solution Θ=0. If there exists a nontrivial solution to Equation (4), i.e., Θ≠0, then the necessary condition is as follows.

(5) $$\frac{d}{d\Theta }\left(\frac{1}{<k>}\sum_{k}kP(k)\frac{\gamma \delta k\tau \Theta (t)}{\gamma k\delta \tau \Theta (t)+\gamma \delta \mu +\gamma k\mu \tau \Theta (t)+\delta \mu k\tau \Theta (t)}\right)\left|\Theta =0\geq 1\right.$$

Namely, $\frac{1}{<k>}\sum_{k}kP(k)\frac{k\tau }{\mu }\geq 1$. Hence the basic reproduction number $R_{0}$ for the psychosocial resonance diffusion of food safety risk under the interaction of consumer heterogeneity and media communication strategies is obtained as follows.

(6) $$R_{0}=\frac{\tau \sum_{k}k^{2}P(k)}{\mu \sum_{k}kP(k)}=\frac{3\varepsilon^{2}e^{-2\sigma \omega }(1-e^{-2\phi })(1-e^{\frac{-\xi^{2}}{3\theta \rho }})\sum_{k}k^{2}P(k)}{2\mu \sum_{k}kP(k)}$$

In scale-free networks, $<k>=2m,<k^{2}>=\sum_{k}k^{2}P(k),P(k)=\frac{2m^{2}}{k^{3}}$, so we assume that the maximum node degree in the network is $k_{\max}$. As the number of network nodes tends to infinity, $k_{\max}=mN^{2}$, and $<k^{2}>=2m^{2}\ln(\frac{k_{\max}}{m})$, and after simplification, the basic reproduction number $R_{0}$ for the psychosocial resonance diffusion of food safety risk is as follows.

(7) $$R_{0}=\frac{3\varepsilon^{2}e^{-2\sigma \omega }(1-e^{-2\phi })(1-e^{\frac{-\xi^{2}}{3\theta \rho }})\sum_{k}k^{2}P(k)}{2\mu \sum_{k}kP(k)}=\frac{3\varepsilon^{2}e^{-2\sigma \omega }(1-e^{-2\phi })(1-e^{\frac{-\xi^{2}}{3\theta \rho }})m\ln(N)}{\mu }$$

#### 2.2.4. Theory Analysis of Psychosocial Resonance Diffusion of Food Safety Risk

**Definition** **1.** *At the beginning of the psychosocial resonance diffusion of food safety risk, the basic reproduction number* $R_{0}$ *is defined as the number of consumers entering an infected state in the spatial–temporal correlation network of food consumers who can infect other susceptible consumers on average before changing to recovered consumers. Typically,* $R_{0}=1$ *can be used as a tipping point for whether the diffusion of psychosocial resonance stops. When* $R_{0}<1$ *, the probability of the psychosocial resonance diffusion of food safety risk is unlikely to sustain chain reactions and will gradually diminish. When* $R_{0}>1$ *, there will be susceptible consumers who will transition to hidden and infected consumers. The larger* $R_{0}$ *is, the higher the diffusion probability in the spatial–temporal correlation network of food consumers.*

**Proposition** **1.** *There is a unique diffusion equilibrium for the psychosocial resonance of food safety risk* $\Theta^{*}$, and $\Theta^{*}=0$.

**Proof of Proposition 1.**  Let us define $W_{k}(\Theta )$, as shown in Equation (8).

(8) $$W_{k}(\Theta )=\frac{k^{2}P(k)}{<k>}\sum_{k}\frac{\gamma \delta k\tau \Theta (t)}{\gamma k\delta \tau \Theta (t)+\gamma \delta \mu +\gamma k\mu \tau \Theta (t)+\delta \mu k\tau \Theta (t)}$$

(9) $$W_{k}^{\prime }(\Theta )=\frac{k^{2}P(k)}{<k>}\sum_{k}\frac{\gamma^{2}\delta^{2}k\tau \mu }{{\left(\gamma k\mu \tau \Theta (t)+\delta k\mu \tau \Theta (t)+\gamma \delta (\mu +k\tau \Theta (t))\right)}^{2}}>0$$

Because of Equation (9) and $W_{k}"(\Theta )<0$, $W_{k}(\Theta )$ is a monotonically increasing concave function about Θ.

(10) $$W_{k}(1)=\frac{k^{2}P(k)}{<k>}\sum_{k}\frac{\gamma \delta k\tau }{\gamma k\delta \tau +\gamma \delta \mu +\gamma k\mu \tau +\delta \mu k\tau }<\frac{kP(k)}{<k>}\sum_{k}\frac{\gamma \delta k\tau }{\gamma k\delta \tau +\gamma \delta \mu +\gamma k\mu \tau +\delta \mu k\tau }=1$$

From Equation (10) and $W_{k}(0)=0$, it is can be concluded that there exists a unique immovable point $\Theta^{*}$ of Equation (8) on [0, 1] and $\Theta^{*}>0$.

Thus, the proof of Proposition 1 is complete. □

It can be seen that when the spatial–temporal correlation network of food consumers converges to a diffusion equilibrium, psychosocial resonance will always exist in the spatial–temporal correlation network of food consumers with a certain probability. That is to say, there will be susceptible, hidden, infected, and recovered consumers in the spatial–temporal correlation network of food consumers at the same time. In this case, consumers should remain vigilant regarding the psychosocial resonance diffusion of food safety risk and avoid being influenced by other consumers and be prepared to deal with psychosocial resonance.

**Proposition** **2.** *Both consumer risk perception level* σ *and consumer risk attention* ω *are negatively correlated with the diffusion threshold of the psychosocial resonance of food safety risk* $R_{0}$*. Consumer sentiment* ϕ *and market noise* ε *are positively correlated with the diffusion threshold of the psychosocial resonance of food safety risk* $R_{0}$.

**Proposition** **3.** *Media report authenticity* θ *and media freedom* ρ *are negatively correlated with the diffusion threshold of the psychosocial resonance of food safety risk* $R_{0}$*. Media report tendency* ξ *is positively correlated with the diffusion threshold of the psychosocial resonance of food safety risk* $R_{0}$.

**Proof Propositions 2 and 3.**  For Equation (7), the first-order derivative with respect to σ can be obtained.

(11) $$\frac{\partial R_{0}}{\partial \sigma }=-\frac{6\omega \varepsilon^{2}e^{-2\sigma \omega }(1-e^{-2\phi })(1-e^{\frac{-\xi^{2}}{3\theta \rho }})m\ln(N)}{\mu }<0$$

The consumer risk perception level is negatively correlated with the diffusion threshold of the psychosocial resonance of food safety risk. Similarly, Equation (12) can be derived.

(12) $$\frac{\partial R_{0}}{\partial \phi }=\frac{6\varepsilon^{2}e^{-2\sigma \omega }e^{-2\phi }(1-e^{\frac{-\xi^{2}}{3\theta \rho }})m\ln(N)}{\mu }>0$$

It can be concluded from Equation (12) that the diffusion threshold of the psychosocial resonance of food safety risk $R_{0}$ is a monotonically increasing function of consumer sentiment φ. In the same way, Equation (13) can be obtained.

(13) $$\frac{\partial R_{0}}{\partial \omega }<0,\frac{\partial R_{0}}{\partial \varepsilon }>0,\frac{\partial R_{0}}{\partial \theta }<0,\frac{\partial R_{0}}{\partial \rho }<0,\frac{\partial R_{0}}{\partial \xi }>0$$

The proof of Propositions 2 and 3 is complete. □

According to Propositions 2 and 3, due to the complex disturbance of consumer connection, it is essential to reduce the probability and scope of the psychosocial resonance diffusion of food safety risk among consumers. This can be achieved by alleviating consumers’ negative sentiment, reducing the noise in the food market, and improving the consumer risk perception level and risk attention. In addition, considering the media communication strategy, media freedom and media report authenticity should be improved when dealing with food safety incidents.

## 3. Results

### 3.1. Analysis of Diffusion Equilibrium Point of Psychosocial Resonance of Food Safety Risk

**Figure 3:**
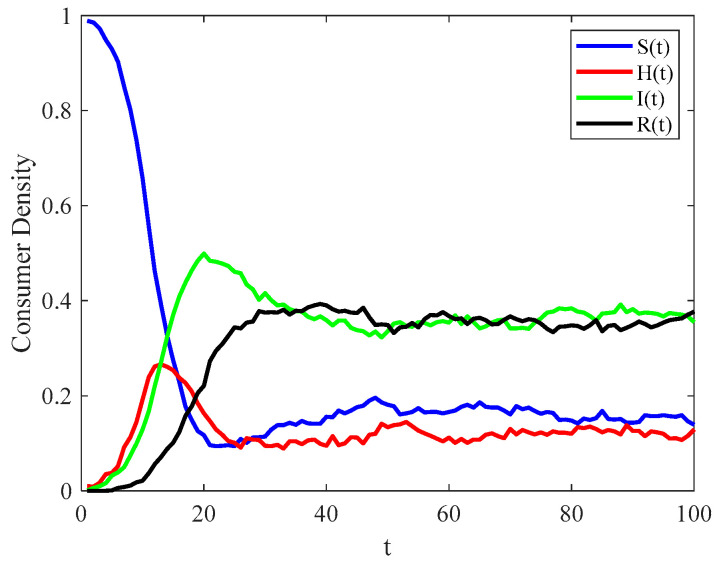
Evolutionary trend in consumer densities in different states in spatial–temporal correlation network of food consumers.

Data on the spatial–temporal correlation network of food consumers is often limited due to the complex interpersonal relationships involved. Therefore, a simulated network is used for analysis to ensure feasibility and generalizability. Suppose there are N consumers in the spatial–temporal correlation network of food consumers. The number of connections of the initial node is $m_{0}=3$, and the number of edges connected when each new node joins is m=3 [64]. At the initial stage, one consumer is in the infected state (I), while the remaining consumers are in the susceptible state (S), N=1000. No consumers are in the hidden H or recovered R state at the initial moment, and s(0)=N−1, h(0)=0, i(0)=1, and r(0)=0. In the real world, m=3 essentially represents the triangular foundation of human social networks. After the emergence of food safety risk, three close contacts, such as family members, friends, merchants, and so on, form a channel for psychosocial resonance diffusion. Similarly, a network with N=1000 is similar to a small-scale social network. In such a network, local panic amplification transforms into psychosocial resonance. By performing calculation and simulation in MATLAB R2022a, referring to the research of Mugnaine et al. [45], with fixed parameters τ=0.2, δ=0.3, μ=0.1, and γ=0.1, the global stability of the diffusion equilibrium point of psychosocial resonance is analyzed, and the evolution characteristics of the scale of consumers in different states is obtained with psychosocial resonance diffusion time t, as shown in Figure 3.

Figure 3 depicts the evolution of consumer densities in different states over time in the spatial–temporal correlation network of food consumers, further verifying the conclusion of Proposition 1. It can be seen from Figure 3 that when food safety incidents break out, the density of susceptible consumers decreases rapidly, then gradually increases before stabilizing eventually. The density of infected consumers and hidden consumers increases rapidly and then decreases gradually after reaching the peak. The density of recovered consumers grows relatively smoothly before stabilizing eventually. During the psychosocial resonance diffusion of food safety risk, even relatively minor food safety risks can spread quickly through the spatial–temporal correlation network of food consumers due to the small-world effect and network clustering. Therefore, the impact of food safety incidents can often spread rapidly to consumers at associated nodes in a short period of time. Affected consumers further disseminate risk to their associated peers, leading to the large-scale cross-regional and cross-population psychosocial resonance of food safety risk. Both hidden and infected consumers show a pattern of increasing, then decreasing, and finally stabilizing. This occurs because hidden consumers have a probability of converting to infected consumers over time. The diffusion speed of the psychosocial resonance of food safety risk gradually slows down after reaching a critical diffusion period. At this time, the scale of consumers in the four states is stable in a fixed range. The density of various consumers in the spatial–temporal correlation network of food consumers tends to be stable in general, with no significant fluctuations thereafter.

**Figure 4:**
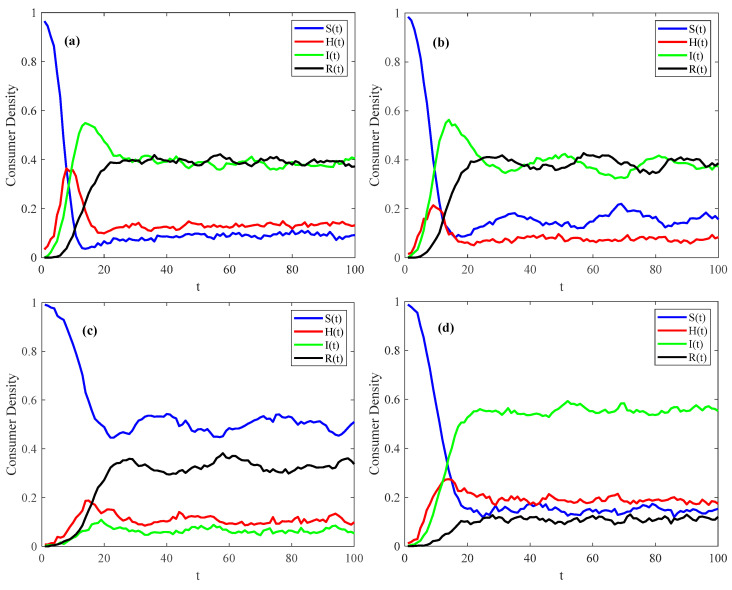
Evolutionary trend in consumer density in different states during psychosocial resonance diffusion of food safety risk under varying mechanism probabilities. (**a**) Increase infection probability; (**b**) Increase conversion probability; (**c**) Increase immune probability; (**d**) Increase immune failure probability.

To better understand the influence of different factors on the psychosocial resonance diffusion of food safety risk in the spatial–temporal correlation network of food consumers, simulations are conducted with varying parameters. This study investigates the diffusion characteristics of the psychosocial resonance of food safety risk under distinct mechanism probabilities (infection probability τ, conversion probability δ, immune probability μ, and immune failure probability γ), as illustrated in Figure 4. The parameter values for numerical simulations are shown in Table 2.

Figure 4 illustrates the evolution trend in the consumer scale in different states during the psychosocial resonance diffusion of food safety risk under varying mechanism probabilities. When the spatial–temporal correlation network of food consumers reaches a steady state, the density of consumers in each state floats in a small range and remains stable on the whole. As shown in Figure 4b, when the conversion probability δ from the hidden to infected state increases while keeping other parameters unchanged, the spatial–temporal correlation network of food consumers reaches a stable state more quickly. Under this condition, the density of hidden consumers decreases, while the density peak and steady-state scale of infected consumers are higher. This shows that an increase in conversion probability δ not only accelerates the stabilization of the spatial–temporal correlation network of food consumers but also enhances the cross-regional and cross-population psychosocial resonance diffusion of food safety risk. In Figure 4d, with immune failure probability γ increasing and other parameters remaining unchanged, when the spatial–temporal correlation network of food consumers reaches a steady state, the density of susceptible consumers does not change significantly, the density of recovered consumers decreases, and the density of hidden and infected consumers increases. This shows that higher immune failure probability will also cause the psychological resonance of food safety risk to spread rapidly among different regions and populations.

As can be seen from Figure 4a, when other parameters remain unchanged, increasing infection probability τ makes the spatial–temporal correlation network of food consumers reach a stable state more quickly. When the spatial–temporal correlation network of food consumers reaches a steady state, the quantity of susceptible consumers decreases, while the quantity of hidden consumers increases. Moreover, during the psychosocial resonance diffusion of food safety risk, both hidden and infected consumers exhibit higher density peaks. This shows that an increase in infection probability can not only make the spatial–temporal correlation network of food consumers reach a stable state faster but also intensify the psychological resonance of food safety risk across different regions. These findings are consistent with the research results of Liang et al. and Mugnaine et al. [45,65] on the impact of infection probability on risk diffusion characteristics. As can be seen from Figure 4c, with an increase in immune probability μ and other parameter values remaining unchanged, the consumer density of each state fluctuates more, while it takes longer for the spatial–temporal correlation network of food consumers to reach a steady state. At this time, when the network reaches a steady state, the density of susceptible consumers increases, while the density of hidden, infected, and recovered consumers decreases. Moreover, during the psychosocial resonance diffusion of food safety risk, the peak value of hidden and infected consumers decreases. This shows that an increase in immune probability not only makes the spatial–temporal correlation network of food consumers reach a steady state more slowly but also controls the spread of the psychological resonance of food safety risk to other areas. However, unlike existing studies that simulate density changes in different states under only a single parameter configuration [45,66], this paper systematically varies the values of the aforementioned four probabilities, as shown in Table 2. This approach makes the effects of these four probabilities on the psychological diffusion of food safety risk more visible. Moreover, while existing research typically employs considerably larger values of N [46,47], this study adopts a relatively smaller value of N, facilitating the observation of subtle variations in density change.

### 3.2. Spatial–Temporal Evolutionary Characteristics of Psychosocial Resonance Diffusion of Food Safety Risk

This subsection aims to simulate and analyze the influence of multiple factors on the psychological resonance diffusion of food safety risk under different parameter values. Therefore, the analysis process focuses on the relative change trend in parameters and the results, rather than focusing on specific numerical values. In order to ensure the reliability and stability of the operation results, the network is simulated 20 times under a fixed value, and the average density value of the simulation results is taken. With reference to the studies of Wang et al. (2019) and Cascante et al. (2022) [48,67], the benchmark parameter values were set as follows: ε=0.7,σ=0.3,ϕ=0.4,ω=0.2,θ=0.2,ρ=0.3,ξ=0.5. Equation (2) was simulated to explore the spatial–temporal evolution characteristics of the psychological resonance diffusion of food safety risk.

#### 3.2.1. Spatial–Temporal Evolutionary Characteristics of Psychosocial Resonance Diffusion of Food Safety Risk Under Influence of Consumer Heterogeneity

In order to describe the spatial–temporal evolution of the psychosocial resonance diffusion of food safety risk under the influence of consumer heterogeneity, simulations were conducted based on different parameter values of the consumer risk perception level σ, consumer sentiment ϕ, consumer risk attention ω, and market noise ε.

**Figure 5:**
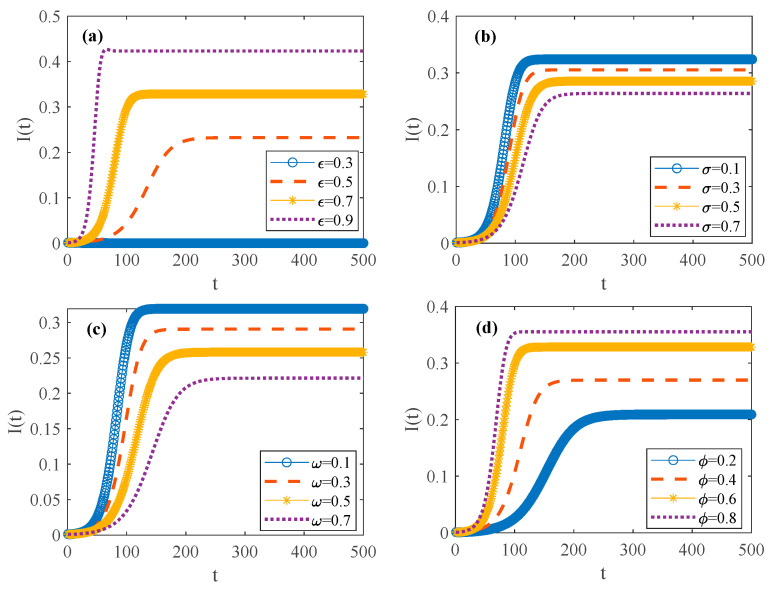
Spatial–temporal evolutionary characteristics of psychosocial resonance diffusion of food safety risk under influence of consumer heterogeneity. (**a**) different values of market noise; (**b**) different values of consumer risk perception level; (**c**) different values of risk attention; (**d**) different values of consumer sentiment.

Figure 5 shows the change in infected consumer density during the psychosocial resonance diffusion of food safety risk, with the varying values of the consumer risk perception level σ, consumer sentiment ϕ, consumer risk attention ω, market noise ε, and other initial conditions remaining unchanged. Under the influence of consumer heterogeneity, after a certain diffusion time, the density of infected consumers eventually tends to a fixed value, and the spatial–temporal correlation network of food consumers reaches a steady state, according to Equation (2). It is worth noting that in Figure 5a, I(t) appears to be zero. This occurs because when market noise is low, infection probability τ is at a low level, resulting in a diffusion threshold of the psychosocial resonance of food safety risk $R_{0}$ less than 1. When the threshold is smaller than 1, psychosocial resonance will not continue to spread in the network. As can be seen from Figure 5a,d, the density of infected consumers increases with the rise in market noise and consumer sentiment. And it shortens the diffusion time for the spatial–temporal correlation network of food consumers to reach the steady state and accelerates the psychosocial resonance diffusion of food safety risk across regions and populations. The reason for this could be that an increase in market noise will make consumers panic and lead to anxiety and so on, resulting in reduced confidence in food safety. Thus, the psychosocial resonance of food safety risk spreads rapidly among different regions and populations. Similarly, when consumer sentiment is negative, consumers tend to perceive food safety risk more acutely, leading to the accelerated diffusion of the psychosocial resonance of food safety risk within the spatial–temporal correlation network of food consumers.

As can be observed from Figure 5b,c, an increase in the consumer risk perception level and consumer risk attention reduces the density of infected consumers in the spatial–temporal correlation network of food consumers and slows down the spread rate. A higher consumer risk perception level can reduce the likelihood of panic and cognitive bias when encountering food safety risks, thereby enabling consumers to make correct consumption decisions. Consequently, this decrease in panic responses limits the further diffusion of the psychosocial resonance of food safety risk in other regions and populations. Moreover, higher consumer risk attention can inhibit risk perception. Meanwhile, a high degree of involvement in food safety risk will drive consumers to understand the trend in food safety risk and relieve risk pressure with self-adjustment, thus effectively reducing the diffusion probability of the psychosocial resonance of food safety risk. These two reasons collectively reduce the density of infected consumers within the spatial–temporal correlation network of food consumers.

#### 3.2.2. Spatial–Temporal Evolutionary Characteristics of Psychosocial Resonance Diffusion of Food Safety Risk Under Influence of Media Communication Strategies

To characterize the spatial–temporal evolution of the psychosocial resonance diffusion of food safety risk under media communication strategies, simulation experiments are performed with parameter configurations reflecting distinct media report authenticity θ, media freedom ρ, and media report tendency ξ values.

**Figure 6:**
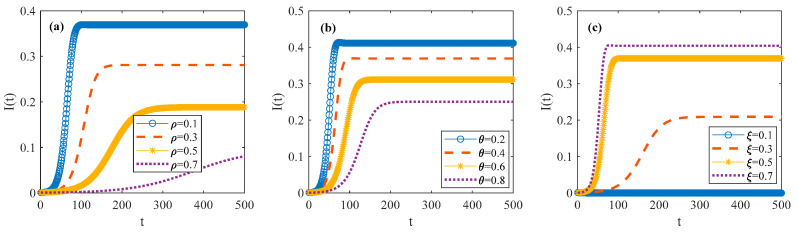
Spatial–temporal evolutionary characteristics of psychosocial resonance diffusion of food safety risk under influence of media communication strategies. (**a**) different values of media freedom; (**b**) different values of media report authenticity; (**c**) different values of media report tendency.

Figure 6 illustrates the variation in the density of infected consumers over the diffusion time of the psychosocial resonance of food safety risk, with unchanged initial conditions and different values of media report authenticity θ, media freedom ρ, and media report tendency ξ. According to Equation (2), under the influence of media communication strategies, the spatial–temporal correlation network of food consumers reaches a steady state after the psychosocial resonance of food safety risk reaches a certain diffusion time. As shown in Figure 6a,b, higher levels of media report authenticity and media freedom correspond to a decrease in the density of infected consumers within the spatial–temporal correlation network of food consumers. Media report authenticity and greater media freedom enhance media oversight and information transparency. Consequently, consumers gain a better understanding of the occurrence and development of food safety incidents, which enables them to identify the psychosocial resonance of food safety risk more quickly and prepare to address it, thereby preventing the further spread of psychosocial resonance to other regions.

Figure 6c reveals that when the media tends to report more negative information, the density of infected consumers I(t) gradually increases from zero. Similarly, this occurs because when media report tendency is low, infection probability τ is at a low level, resulting in a diffusion threshold of the psychosocial resonance of food safety risk $R_{0}$ less than 1. When the threshold is greater than 1, psychosocial resonance will continue to spread in the network. When the media leans more toward negative reporting, consumers are more easily influenced by media reports, which amplifies food safety risk perception and reduces public trust in food safety, leading to panic in the food market. At this point, the psychosocial resonance of food safety risk rapidly spreads through the spatial–temporal correlation network of food consumers, affecting other consumers within the network. Negative reports lead to the accumulation and diffusion of food safety risk within the spatial–temporal correlation network of food consumers, increasing the extent and scope of the psychosocial resonance of food safety risk across regions and populations. Consequently, the steady-state density of infected consumers rises.

#### 3.2.3. Spatial–Temporal Evolutionary Characteristics of Psychosocial Resonance Diffusion of Food Safety Risk Under Interaction of Consumer Heterogeneity and Media Communication Strategies

In prior studies, most scholars have focused on simulating and analyzing the evolutionary characteristics described above [45,46,47], with limited attention given to simulating the diffusion threshold. Therefore, this paper subsequently simulates and analyzes diffusion thresholds, enabling a more intuitive identification of the conditions under which the psychosocial resonance of food safety risk is more likely to occur and propagate. This subsection further analyzes the spatial–temporal evolutionary characteristics of the psychosocial resonance of food safety risk under the interaction of consumer heterogeneity and media communication strategies.

**Figure 7:**
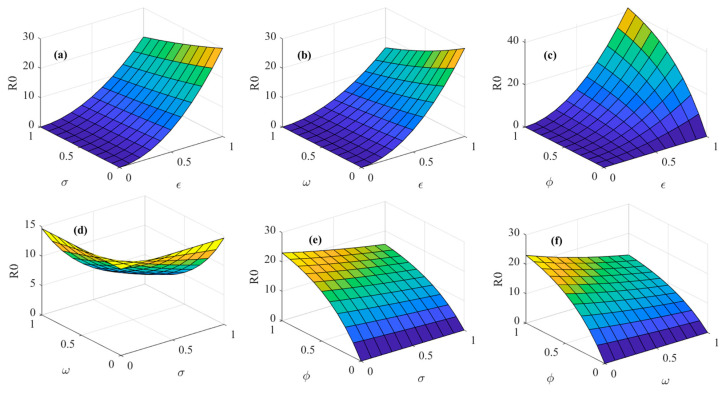
Spatial–temporal evolutionary characteristics of psychosocial resonance diffusion of food safety risk under interaction of consumer heterogeneity. (**a**) The interaction between market noise and consumer risk perception level; (**b**) The interaction between market noise and consumer risk attention; (**c**) The interaction between market noise and consumer sentiment; (**d**) The interaction between consumer risk attention and consumer risk perception level; (**e**) The interaction between consumer sentiment and consumer risk perception level; (**f**) The interaction between consumer risk attention and consumer sentiment.

Figure 7 illustrates the effects of consumer heterogeneity factors on the psychosocial resonance diffusion of food safety risk, with initial conditions held constant. As shown in Figure 7a,b, when market noise ε interacts with the consumer risk perception level σ and consumer risk attention ω, the diffusion threshold of the psychosocial resonance of food safety risk decreases with an increase in the consumer risk perception level σ and consumer risk attention ω but increases with an increase in market noise ε. From Figure 7c, it can be seen that under the interaction of market noise ε and consumer sentiment ϕ, the diffusion threshold of the psychosocial resonance of food safety risk increases with an increase in market noise ε and consumer sentiment ϕ, and the threshold change becomes more pronounced when the values of market noise ε and consumer sentiment ϕ are larger. This reveals the complexity of the interaction between consumer sentiment and market noise. In environments with significant market noise, negative emotions are more likely to cause information overload or cognitive biases among individuals, thereby intensifying the psychosocial resonance diffusion of food safety risk. Similarly, as shown in Figure 7e,f, when consumer sentiment ϕ interacts with the consumer risk perception level σ and consumer risk attention ω, the diffusion threshold of the psychosocial resonance of food safety risk increases with an increase in consumer sentiment ϕ but decreases with an increase in the consumer risk perception level σ and consumer risk attention ω. From Figure 7d, it can be observed that under the interaction of the consumer risk perception level ω and consumer risk attention ω, the diffusion threshold of the psychosocial resonance of food safety risk decreases as the values of the consumer risk perception level σ and consumer risk attention ω increase. This indicates that the consumer risk perception level and consumer risk attention are pivotal factors in controlling the diffusion of psychosocial resonance. A simultaneous increase in both the consumer risk perception level and consumer risk attention can suppress the contagion of psychosocial resonance within the spatial–temporal correlation network of food consumers and lower the diffusion threshold. Therefore, based on Figure 7, it can be concluded that when facing food safety risks, consumers should enhance their risk perception level and attention paid to risks. This not only helps individual consumers make more rational judgments, but more importantly, it can effectively reduce the risk of consumer groups falling into irrationality and amplifying psychosocial resonance and enhance the resilience of risk resistance at the group level. At the same time, consumers should pay attention to adjusting their sentiments and be alert to the existence of market noise. They should try to avoid being influenced by consumers who have already been infected with psychosocial resonance and cut off the transmission path of the negative resonance chain.

**Figure 8:**
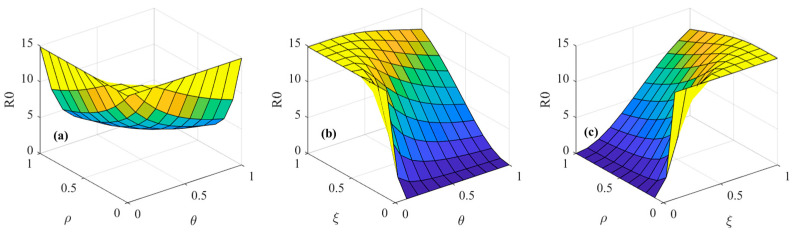
Spatial–temporal evolutionary characteristics of psychosocial resonance diffusion of food safety risk under interaction of media communication strategies. (**a**) The interaction between media freedom and media report authenticity; (**b**) The interaction between media report tendency and media report authenticity; (**c**) The interaction between media report tendency and media freedom.

Figure 8 illustrates the impact of multiple media communication strategy factors on the psychosocial resonance diffusion of food safety risk under unchanged initial conditions. As shown in Figure 8a, under the interaction of media report authenticity θ and media freedom ρ, as both media report authenticity θ and media freedom ρ increase, the diffusion threshold of the psychosocial resonance of food safety risk shows a smooth downward trend. From Figure 8b,c, it can be seen that when media report tendency ξ interacts with media report authenticity θ and media freedom ρ, the diffusion threshold of the psychosocial resonance of food safety risk slightly decreases with a rise in media report authenticity θ and media freedom ρ but significantly increases with a rise in media report tendency ξ. It is evident that the risk-enhancing effect of media report tendency ξ significantly outweighs the risk-mitigating effects of media report authenticity θ and media freedom ρ. Therefore, according to Figure 8, it can be concluded that in the face of food safety incidents, the media should enhance the authenticity of their reports and publish more objective and positive reports. Meanwhile, the government should take corresponding measures to ensure media freedom, thereby effectively controlling the cross-regional diffusion of the psychosocial resonance of food safety risk.

**Figure 9:**
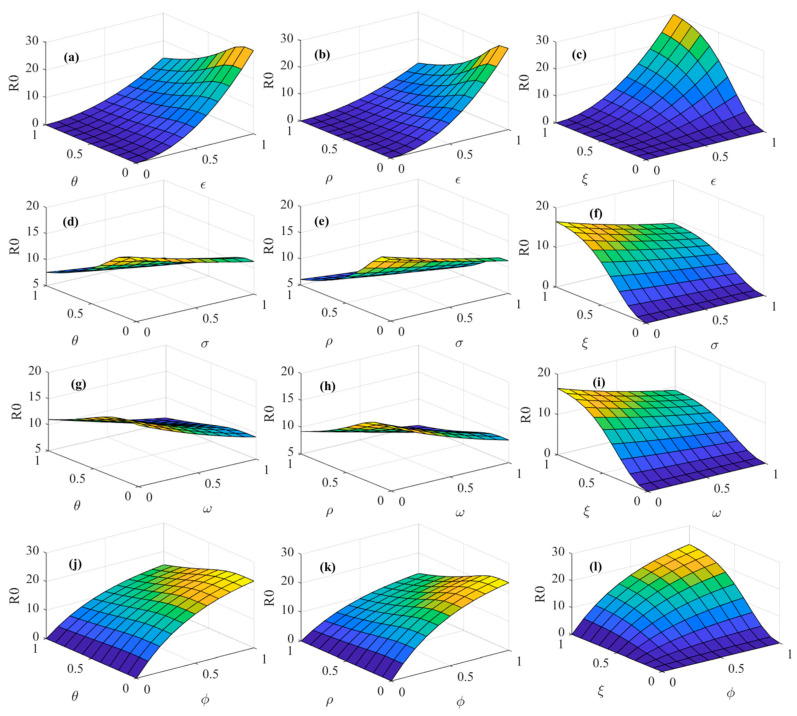
Spatial–temporal evolutionary characteristics of psychosocial resonance diffusion of food safety risk under interaction of consumer heterogeneity and media communication strategies.

Figure 9 shows the effects of the interaction of consumer heterogeneity and media communication strategies on the psychosocial resonance diffusion of food safety risk while keeping other initial conditions constant. From Figure 9a–c, it is observable that when market noise ε interacts with media report authenticity θ, media freedom ρ, and media report tendency ξ, the diffusion threshold of the psychosocial resonance of food safety risk increases with the growth of market noise ε and media report tendency ξ and decreases slightly with an enhancement in media report authenticity θ and media freedom ρ. This suggests that regulating market noise ε and media report tendency ξ at the same time can effectively control the psychosocial resonance diffusion of food safety risk in the spatial–temporal correlation network of food consumers.

As shown in Figure 9d,e, the diffusion threshold of the psychosocial resonance of food safety risk decreases with an increase in the consumer risk perception level σ, media report authenticity θ, and media freedom ρ when the consumer risk perception level σ interacts with media report authenticity θ and media freedom ρ. Comparing Figure 9g,h, when consumer risk attention ω interacts with media report authenticity θ and media freedom ρ, the diffusion threshold of the psychosocial resonance of food safety risk decreases with an increase in consumer risk attention ω, media report authenticity θ, and media freedom ρ.

Comparing Figure 9f,i, it is noticeable that an increase in media report tendency ξ increases the diffusion threshold of the psychosocial resonance of food safety risk, but increases in the consumer risk perception level σ and consumer risk attention ω lower the diffusion threshold. When media report tendency ξ interacts with the consumer risk perception level σ and consumer risk attention ω, the threshold undergoes gradual changes within a confined range overall. Comparing Figure 9f,j,k, it can be concluded that when consumer sentiment ϕ interacts with media report authenticity θ, media freedom ρ, and media report tendency ξ, the diffusion threshold of the psychosocial resonance of food safety risk decreases slightly with an increase in media report authenticity θ and media freedom ρ and increases significantly with a rise in consumer sentiment ϕ and media report tendency ξ. Moreover, the diffusion threshold of the psychosocial resonance of food safety risk tends to increase significantly when both media report tendency ξ and consumer sentiment ϕ increase at the same time. This suggests that the strengthening effect of consumer sentiment ϕ on the diffusion threshold of the psychosocial resonance of food safety risk is greater than the weakening effect of media freedom ρ and media report authenticity θ on the diffusion threshold of the psychosocial resonance of food safety risk. In addition, by comparing Figure 9 and Figure 8, media report tendency ξ has a relatively small impact on the psychosocial resonance diffusion of food safety risk when acting alone, while the change in the diffusion threshold of the psychosocial resonance of food safety risk is relatively more pronounced as media report tendency ξ interacts with consumer heterogeneity factors. In other words, the single factor of media report tendency ξ has a limited impact on the psychosocial resonance of food safety risk, but the negative impact of media report tendency ξ on consumer groups and food markets is amplified by consumer sentiment ϕ and market noise ε, which exacerbates the extent of the psychosocial resonance diffusion of food safety risk in different regions. Negative media reports are likely to trigger cross-regional and cross-population chain reactions within a short period of time, thus creating a wider psychosocial resonance of food safety risk. Therefore, in controlling the psychosocial resonance diffusion of food safety risk, it is necessary to focus on controlling the influence of media report tendency and market noise on consumers, as well as enhancing the consumer risk perception level and consumer risk attention. Moreover, differentiated risk communication strategies should be adopted according to the conditions of food markets and consumer characteristics in different regions so as to effectively control the speed and scale of the cross-regional spread of the psychosocial resonance of food safety risk.

### 3.3. Robustness Test

**Figure 10:**
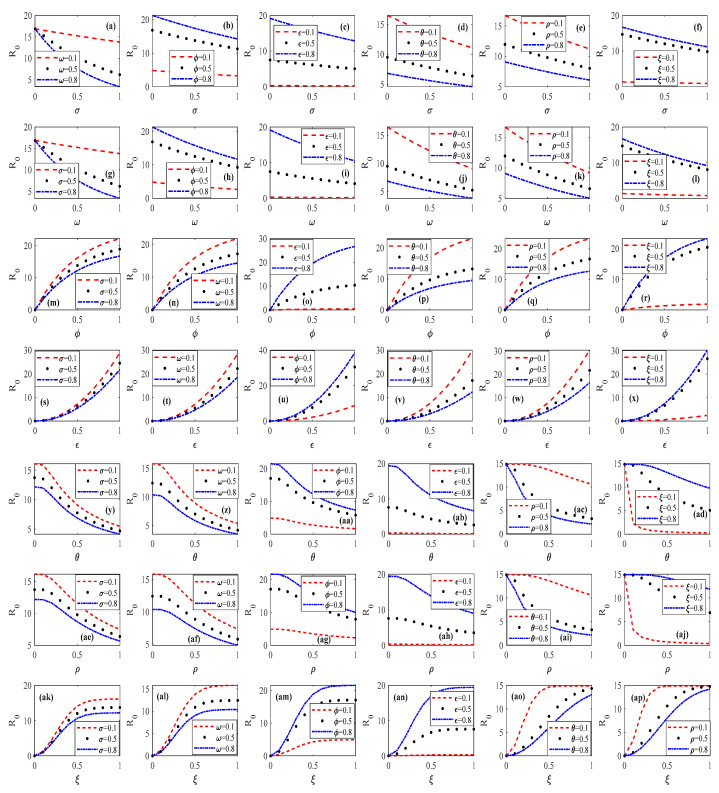
Robustness test for psychosocial resonance diffusion of food safety risk under interaction of consumer heterogeneity and media communication strategies.

To test the robustness of the psychosocial resonance diffusion of food safety risk under the interaction of consumer heterogeneity and media communication strategies, this paper changes the values of the consumer heterogeneity factor and media communication strategy factor and then analyzes the magnitude of the change in the scale of the psychosocial resonance diffusion of food safety risk. The results are shown in Figure 10. As can be seen from Figure 10, the impact of the same parameter on the evolutionary trend in the psychosocial resonance diffusion of food safety risk is the same as that analyzed above under different combinations of consumer heterogeneity factors and media communication strategy factors.

In the robustness test results, each row of figures represents the experimental results obtained by holding one of the following parameters constant while varying the remaining parameters: consumer risk perception level σ, consumer risk attention ω, consumer sentiment ϕ, market noise ε, media report authenticity θ, media freedom ρ, or media report tendency ξ. Consequently, this combinatorial testing approach generated a total of 72 distinct graphical outputs. Figure 10a–f display the results with the consumer risk perception level σ held constant and other parameters systematically varied. These plots demonstrate that consumer risk attention ω, media report authenticity θ, and media freedom ρ consistently suppress the diffusion threshold $R_{0}$, whereas consumer sentiment ϕ, market noise ε, and media report tendency ξ enhance the threshold. Figure 10g–l present the outcomes with consumer risk attention ω fixed and other parameters modulated. This confirms that the consumer risk perception level σ, media report authenticity θ, and media freedom ρ inhibit the diffusion threshold, while consumer sentiment ϕ, market noise ε, and media report tendency ξ amplify it. By analogy, the final result is highly consistent with the results of the simulation analysis mentioned above. This indicates that the conclusions obtained from the simulation in this paper are highly robust. In addition, the results presented in Figure 10 can effectively prove the research conclusions obtained in Figure 7, Figure 8 and Figure 9 and further highlight the robustness of the model proposed in this paper and the simulation results.

## 4. Conclusions

This paper constructs a CA-SHIRS model of the psychosocial resonance of food safety risk, taking into account consumer heterogeneity and media communication strategies and combining it with spatial–temporal dynamic characteristics. On this basis, this paper analyzes the diffusion mechanism of the psychosocial resonance of food safety risk by using the basic reproduction number and reveals the dynamic evolution law of the psychosocial resonance of food safety risk in the space-time dimension. The main conclusions obtained in this paper are as follows:

(1) With respect to different mechanism probabilities and the psychosocial resonance of food safety risk, increasing infection probability and immune failure probability not only allows for faster risk contagion in the spatial–temporal correlation network of food consumers but also enlarges the scale of the psychosocial resonance diffusion of food safety risk. Increasing conversion probability increases the peak and steady-state densities of infected consumers, exacerbating the scale of the psychosocial resonance diffusion of food safety risk in the network. Increasing immune probability reduces the scale of the psychosocial resonance diffusion of food safety risk and slows down the psychosocial resonance diffusion of food safety risk in the spatial–temporal correlation network of food consumers. Additionally, in the CA-SHIRS model of psychosocial resonance diffusion, once the diffusion reaches a certain time point, the steady-state densities of consumers in each state fluctuate slightly within a fixed range, and the overall trend stabilizes.

(2) In terms of consumer heterogeneity and the psychosocial resonance of food safety risk, the diffusion threshold of the psychosocial resonance of food safety risk is positively correlated with market noise and consumer sentiment and negatively correlated with the consumer risk perception level and consumer risk attention. To some extent, market noise amplifies the impact of consumer sentiment on the psychosocial resonance diffusion of food safety risks. Regarding media communication strategies and the psychosocial resonance of food safety risk, the diffusion threshold of the psychosocial resonance of food safety risk is negatively correlated with media report authenticity and media freedom and positively correlated with media report tendency.

(3) In terms of the interaction between consumer heterogeneity and media communication strategies, when media report tendency interacts with consumer sentiment and market noise, market noise and consumer sentiment amplify the impact of media report tendency on the psychosocial resonance of food safety risk. This, in turn, reinforces the cross-crowd, cross-regional diffusion of the psychosocial resonance of food safety risk. In addition, when consumer sentiment interacts with media report authenticity and media freedom, consumer sentiment enhances the spread of the psychosocial resonance of food safety risk across populations and regions. Furthermore, the risk-enhancing effect of consumer sentiment is stronger than the risk-weakening effect of media report authenticity and media freedom.

The findings of this paper can offer theoretical reference for food regulatory authorities to prevent the psychosocial resonance diffusion of food safety risk and maintain food market stability. However, this paper only investigates the mechanism and spatial–temporal evolutionary characteristics of the psychosocial resonance diffusion of food safety risk, without conducting research on the control of the psychosocial resonance of food safety risk. Further studies should explore how to effectively control the psychosocial resonance diffusion of food safety risk from the perspective of the risk immunization mechanisms of food enterprises and the government’s regulatory strategy.

In summary, this study has significant theoretical and practical implications. Theoretically, it innovatively integrates cellular automata (CA) with the SHIRS model to optimize existing epidemic models applied to food safety risk research. Simultaneously, this paper reveals synergistic effects between media communication strategies and consumer heterogeneity by incorporating the space-time perspective, consumer heterogeneity, and media communication strategy into a unified research framework. Practically, the conclusions of this paper can provide actionable insights for food regulatory authorities and media entities to control the spread of the psychosocial resonance of food safety risk and maintain the stability of the food market. However, this paper only studies the diffusion mechanism and spatiotemporal evolution characteristics of the psychosocial resonance of food safety risk but does not study the control of the psychosocial resonance of food safety risk. Future research can further focus on the immunity mechanism of food enterprises and government supervision strategies and how to effectively control the spread of the psychosocial resonance of food safety risk.

## Figures and Tables

**Table 1:** Descriptions and numerical values of model parameters.

Parameters	Descriptions	Baseline Values	Value Ranges
τ	Infection probability	0.2	[0, 1]
δ	Conversion probability	0.3	[0, 1]
μ	Immune probability	0.1	[0, 1]
γ	Immune failure probability	0.1	[0, 1]
N	Total quantity of consumers in the network	1000	Positive Integer
m	Number of edges connected when each new node joins	3	Positive Integer
$m_{0}$	Number of connections of the initial node	3	Positive Integer
σ	Consumer risk perception level	0.3	[0, 1]
ϕ	Consumer sentiment	0.4	[0, 1]
ω	Consumer risk attention	0.2	[0, 1]
ε	Market noise	0.7	[0, 1]
θ	Media report authenticity	0.2	[0, 1]
ρ	Media freedom	0.3	[0, 1]
ξ	Media report tendency	0.5	[0, 1]

**Table 2:** Numerical variations in CA-SHIRS model of psychosocial resonance diffusion of food safety risk under varying mechanism probabilities.

τ	δ	μ	γ	Parameter Variation	Trend Chart
0.2	0.3	0.1	0.1	Remain unchanged	Figure 3
0.5	0.3	0.1	0.1	Increase infection probability	Figure 4a
0.2	0.5	0.1	0.1	Increase conversion probability	Figure 4b
0.2	0.3	0.5	0.1	Increase immune probability	Figure 4c
0.2	0.3	0.1	0.5	Increase immune failure probability	Figure 4d

## Data Availability

The original contributions presented in the study are included in the article. Further inquiries can be directed to the corresponding author.
